# Dynamic Control of Topological Defects in Artificial Colloidal Ice

**DOI:** 10.1038/s41598-017-00452-w

**Published:** 2017-04-05

**Authors:** A. Libál, C. Nisoli, C. Reichhardt, C. J. Olson Reichhardt

**Affiliations:** 1 Faculty of Mathematics and Computer Science, Babeş-Bolyai University, Cluj, 400084 Romania; 2 Theoretical Division and Center for Nonlinear Studies, Los Alamos National Laboratory, Los Alamos, New Mexico 87545 USA

## Abstract

We demonstrate the use of an external field to stabilize and control defect lines connecting topological monopoles in spin ice. For definiteness we perform Brownian dynamics simulations with realistic units mimicking experimentally realized artificial colloidal spin ice systems, and show how defect lines can grow, shrink or move under the action of direct and alternating fields. Asymmetric alternating biasing forces can cause the defect line to ratchet in either direction, making it possible to precisely position the line at a desired location. Such manipulation could be employed to achieve mobile information storage in these metamaterials.

## Introduction

Systems mimicking the behavior of spin ice have been studied experimentally and theoretically for nanomagnetic islands^1–11^, superconducting vortices^12–14^, and paramagnetic colloidal particles on photolithographically etched surfaces^13, 15–17^. In each of these particle-based artificial ice systems, the collective lowest energy state is embedded into an ice-manifold where all vertices obey the “2-in/2-out” ice rule: two particles are close to each vertex and two are far from it. It is possible to write information into such a manifold by using an MFM tip^11^ or an optical tweezer^15^ to generate topological defects in the ground state arrangement of the spins. These defects consist of vertices that violate the ice rule and correspond to 3-in/1-out or 3-out/1-in configurations. In magnetic spin ices, such defects are called magnetic monopoles^18^. In colloidal artificial ice, the defects are not magnetically charged but they still carry a topological charge^19^. This implies that they can only appear in pairs separated by a line of polarized ice-rule vertices, and disappear by mutual annihilation. In a square ice geometry, such defect lines are themselves excitations and thus possess a tensile strength^20, 21^ that linearly confines the topological charges and can drive them to mutual annihilation, restoring the ground state configuration.

In this paper we show how an additional biasing force can be used to stabilize, control, and move defect lines written on the ordered ground state of a square colloidal artificial spin ice system. To make contact with recent experimental realizations of this system^15, 22^, we employ a gravitational bias that can be implemented experimentally by tilting the effectively two-dimensional (2D) sample. We consider the interplay of two completely separate control parameters: the tilt that controls the biasing and the perpendicular magnetic field that controls the inter-particle repulsive magnetic forces, as in ref. 15. Adjusting these parameters gives us precise control over the energetics of the system and makes it possible to control the speed of the shrinking or expansion of a defect line. Then, using asymmetrical ac biasing forces and taking advantage of the different mobility of the 1-in and 3-in defects in colloidal ice, we show that the defect line can be made to ratchet, or undergo a net dc motion, in the direction of either of its ends.

The control introduced by the biasing force permits locally stored, compact information to be written into the artificial ice metamaterial by a globally applied force, making it possible to create effective information storage since the write/read heads need to be situated only at the edge of the memory block. Also, by moving localized packets of information with a global force, it is possible to parallelize the information storage and retrieval procedures, increasing the speed in both cases.

## Results

### Model and its simulation

**Figure 1:**
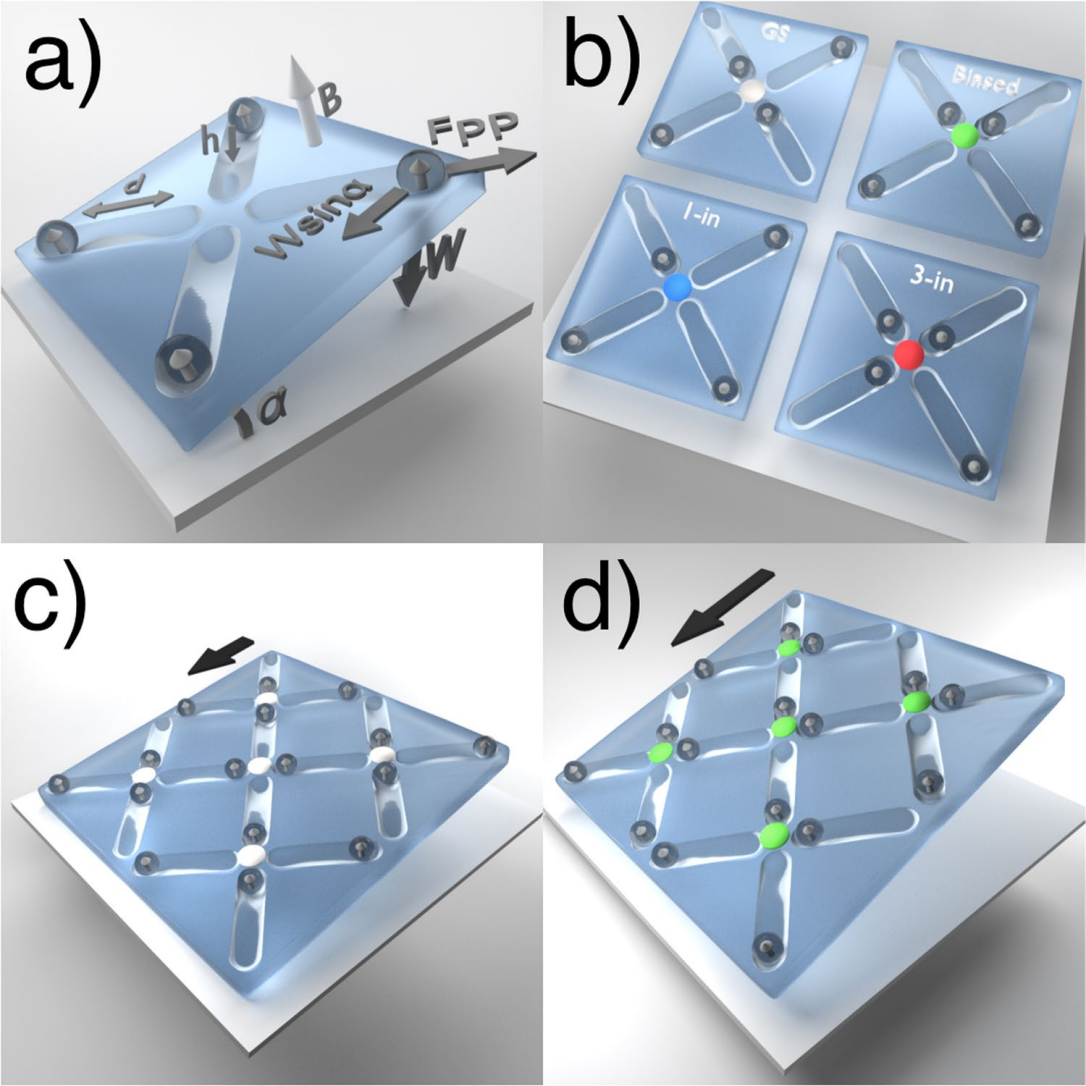
Schematics of the system. (**a**) A single vertex is surrounded by four double-well pinning sites. Labels indicate the distance *d* between the minima, the barrier height *h*, the biasing tilt angle *α*, the magnetic field *B*, the magnetization *m* it induces in the paramagnetic particles, and the pairwise magnetic repulsive forces *F*_*pp*_ acting in the sample plane. *W* is the weight of the particle, and the tangential component *W* sin(*α*) serves as a biasing force. (**b**) Illustration of four possible vertex arrangements with a nonphysical color placed at the vertex center to indicate the vertex type. Ground state (GS, gray), biased state (green), 1-in state (blue), and 3-in state (red). (**c**) The unbiased ground state (gray) in a small segment of the sample for a small bias *α*. (**d**) The biased ground state (green) in a small segment of the sample for a large bias *α*.

In Fig. 1, we show schematics of our system illustrating the interplay between the interparticle and biasing forces. The four pinning sites in Fig. 1(a) represent photolithographically etched grooves in the surface, each of which acts as a gravitational double well with a distance of *d* = 10 *μ*m between the two minima. At the center of the pinning site is a barrier of height *h* = 0.87 *μ*m. Four paramagnetic colloidal particles are each trapped in the gravitational wells by the combination of their own apparent weight (*W* = (*ρ* − *ρ*_liquid_)*gV*) and the normal force from the wall, where *ρ* is the density and *V* is the volume of an individual particle, *ρ*_liquid_ is the density of the surrounding liquid, and *g* is the gravitational constant. A biasing force is introduced by tilting the whole ensemble by *α* degrees with respect to the horizontal. This creates a biasing force *W* sin(*α*) equal to the tangential projection of the apparent weight of the particles, providing us with two independent external tuning parameters: the tilt of the surface and the external magnetic field.

The direction of the external magnetic field $\mathop{B}\limits^{\longrightarrow}$ is indicated by a light arrow in Fig. 1(a). This field is always perpendicular to the sample plane, and it induces magnetization vectors $\vec{m}\propto \vec{B}$ parallel to itself in each of the paramagnetic particles. As a result, the particles repel each other with an isotropic force *F*_*pp*_ ∝ *B* ^2^/*r* ^4^ that acts in the plane. This favors arrangements in which the particles maximize their distance from each other. For an isolated vertex the lowest energy configuration is the 4-out arrangement shown in Fig. 1(a); however, in a system of many coupled vertices, such an arrangement places an occupancy burden on the neighboring vertices. As a result, a multiple-vertex arrangement stabilizes in the low energy ice-rule obeying state illustrated in Fig. 1(c) that is composed of 2-in and 2-out ground state vertices. The four vertex types we observe are shown in Fig. 1(b), where the ground state vertex is colored gray, the biased ice-rule obeying vertex is green, the 1-in vertex is blue, and the 3-in vertex is red. The 1-in and 3-in monopole states carry an extra magnetic charge and serve as the starting and termination vertices for defect lines. It is also possible for 0-in [Fig. 1(a)] and 4-in (not shown) vertices to form, but they are highly energetically unfavorable and do not play a role in our defect line study. For small bias (small *α*), the ground state vertex arrangement of Fig. 1(c) is favored, while for large enough *α*, the system switches to the biased 2-in/2-out arrangement shown in Fig. 1(d).

### Defect line motion

**Figure 2:**
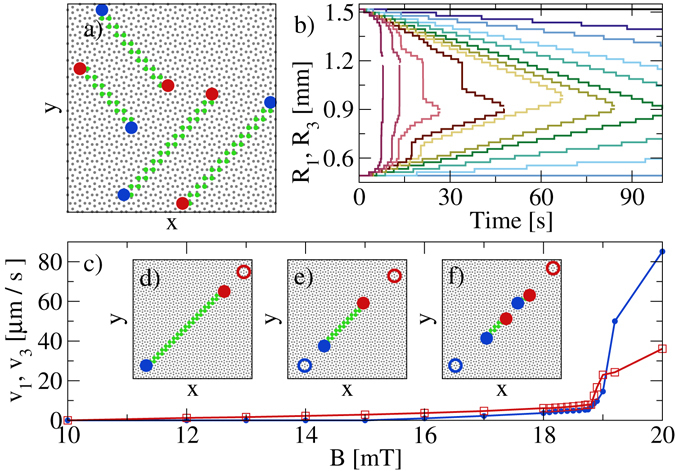
Defect line images and motion. (**a**) The four possible arrangements of the defect lines in a portion of the sample. Red: 3-in vertex; blue: 1-in vertex; green: biased ground state vertex; gray: unbiased ground state vertex. (**b**) The position *R*_1_ of the 1-in (bottom lines) and *R*_3_ of the 3-in (top lines) ends of a defect line vs time at magnetic fields *B* = 20, 19.2, 19, 18.85, 18.8, 18.5, 18, 17, 16, 14, 12, and 10 mT, from left to right. (**c**) The velocity *v*_1_ (blue) and *v*_3_ (red) of the defect ends calculated with a linear fit vs *B*. (**d**–**f**) Illustrations of the different modes of defect line contraction in a portion of the sample. Open circles indicate the original positions of the 3-in and 1-in ends, while closed circles show the final positions. (**d**) Contraction of only the 3-in end. (**e**) Contraction of both ends. (**f**) Contraction of both ends accompanied by nucleation of new defect vertices along the defect line.

Using a 50 × 50 vertex square spin ice sample containing 5000 pinning sites and particles, we initialize the system in the ground state by placing the particles inside the appropriate substrate minima. We then perturb this ground state by introducing a defect line to it, achieved by flipping the effective spins along a diagonal line connecting neighboring vertices. The defect line is composed of a pair of 1-in and 3-in vertices connected by a series of biased ground state vertices. All four possible orientations of the defect line are illustrated in Fig. 2(a). We focus on the dynamics of the defect line in the center of the panel; all other lines show the same behavior when the direction along which the tilt *α* is applied is rotated appropriately.

In Fig. 2(b), to illustrate the contraction of the defect line in the absence of a biasing force when magnetic fields *B* of different strengths are applied to the system, we plot the positions ${R}_{1}=\sqrt{{x}_{1}^{2}+{y}_{1}^{2}}$ and ${R}_{3}=\sqrt{{x}_{3}^{2}+{y}_{3}^{2}}$ of the endmost 1-in and 3-in vertices, respectively, as a function of time. We can distinguish several stages of the contraction process. For very low *B*, particle-particle interactions are very weak and the defect line remains static, as shown by the constant values of *R*_1_ and *R*_3_ for *B* = 10 mT. For small fields in the range of 12 mT < *B* < 16 mT, the 3-in end of the defect contracts while the 1-in end of the defect remains static, as shown for *B* = 12 and 14 mT. For 16 mT ≤ *B* ≤ 18.5 mT, both ends of the defect contract, as illustrated for *B* = 16, 17, 18, and 18.5 mT. For *B* > 18.5 mT, the defect line cannot contract as fast as the rate dictated by the field, and as a result the line breaks up into 1-in/3-in vertex pairs along its length. In a narrow range of fields just above 18.5 mT, pair formation occurs only near the lower mobility 1-in end of the defect line, since only this end of the line cannot keep up with the contraction speed. At slightly higher fields, the 3-in end of the defect line also lags behind the contraction speed and nucleation of 1-in/3-in pairs occurs along the whole length of the line. In this regime, the defect line shrinks by eliminating the small lines into which it has broken instead of by a step-by-step contraction along its length. Nucleation events that occur close to the end of the line induce sudden large jumps in *R*_1_ and/or *R*_3_ in Fig. 2(b). Here, the shrinking process eliminates the short segment at the end of the defect line, causing a sudden change in identity of the endmost vertex, which shifts the location of *R*_1_ or *R*_3_ to the end of the surviving portion of the defect line. The jumps follow the nucleation events after a delay corresponding to the amount of time required for the endmost small line to annihilate by having its two ends contact each other.

We determine the velocity *v*_1(3)_ of the two defect ends from a linear fit of the *R*_1(3)_(*t*) curves, and plot *v*_1_ and *v*_3_ versus *B* in Fig. 2(c). For *B* < 15 mT only the 3-in end moves, as illustrated in Fig. 2(d). Both ends are mobile for 15 mT ≤ *B* ≤ 18.5 mT, but *v*_3_ > *v*_1_, as shown in Fig. 2(e). For *B* > 18.5 mT, defect line fracturing and spontaneous 1-in/3-in pair creation along the defect line occur, as illustrated in Fig. 2(f).

Naively one would expect both ends of the defect line to have the same mobility, *v*_1_ = *v*_3_, as occurs in magnetic spin ices. To understand the difference between *v*_1_ and *v*_3_, note that although in magnetic spin ice the 1-in and 3-in vertices have the same energy, in colloidal spin ice they do not. In dipolar magnetic artificial spin ice^8^, frustration occurs at the vertex level and consists of a frustration of the pairwise interaction. In contrast, in colloidal spin ice the frustration is a collective effect arising from the fact that topological charge conservation prevents vertices from adopting the lowest single-vertex energy configurations, the 0-in or 1-in states^19^. Thus *the colloidal ice-manifold is composed of vertices that are not, by themselves, the lowest energy vertices, yet that produce the lowest energy manifold* ^19^.

To illustrate this point, in Table 1 we list the energy of each possible vertex configuration in our colloidal spin ice at an external field of *B* = 16 mT. The table shows that for the defect line to shrink by moving its 1-in end, the 1-in vertex must undergo an energetically unfavorable transformation into a ground state vertex while a biased vertex makes an energetically favorable transformation into a 1-in vertex. In contrast, when the 3-in end moves, a 3-in vertex undergoes an energetically favorable transformation into a ground state vertex while a biased vertex makes an energetically unfavorable transition to a 3-in vertex. The total energy gain is equal to the transformation energy of changing a biased vertex into a ground state vertex in each case, but the initiating transition is energetically favorable for the 3-in end and unfavorable for the 1-in end, so that *v*_3_ > *v*_1_.

**Table 1:** Magnetostatic energy for each vertex type at *B* = 16 mT.

Vertex Type	Particle Configuration	Energy [10^−18^ *J*]
0-in	0 0 0 0 (×1)	10.007
1-in	0 0 0 1 (×4)	15.568
ground state	0 1 0 1 (×2)	24.727
biased 2-in	0 0 1 1 (×4)	32.905
3-in	0 1 1 1 (×4)	53.837
4-in	1 1 1 1 (×1)	86.542

An example configuration for each vertex is listed. “1” (“0”) indicates a colloid close to (far from) the vertex and (×*n*) indicates that *n* different equivalent configurations can be obtained by rotation.

**Figure 3:**
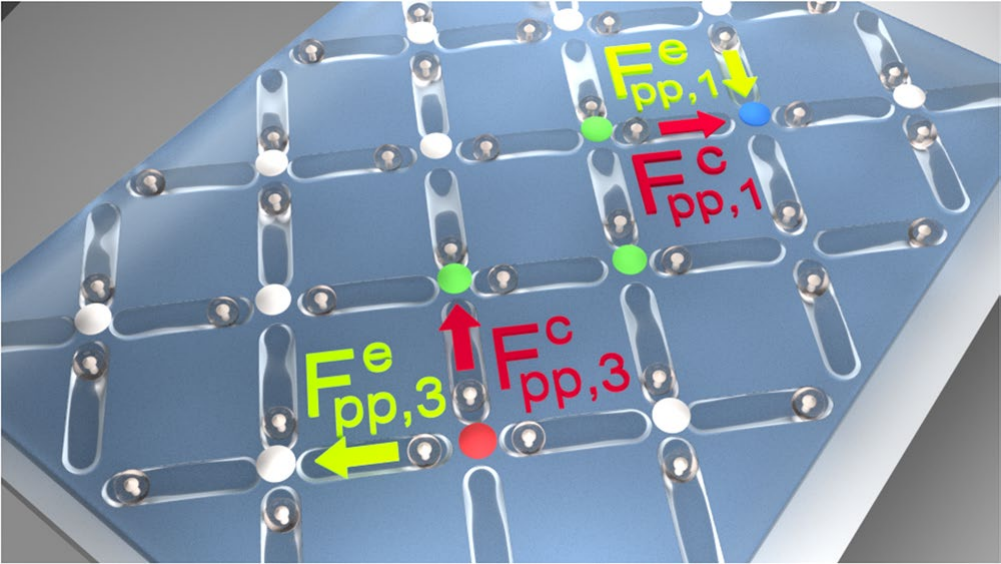
Schematic showing the forces that are responsible for contracting and extending the defect line. The 1-in and 3-in ends of the line are marked blue and red, respectively, while the biased ground state vertices along the defect line are marked green. Particle-particle forces that act to extend (e, green lettering and arrows) or contract (c, red lettering and arrows) the defect are marked for the 1-in end, ${F}_{pp,1}^{e}$ and ${F}_{pp,1}^{c}$, and for the 3-in end, ${F}_{pp\mathrm{,3}}^{e}$, ${F}_{pp,3}^{c}$.

Taking into account the overdamped dynamics of the system, the mechanism for the asymmetry in *v*_1_ and *v*_3_ can be understood more clearly by considering the forces acting on an individual particle. During the transition of the particle from one trap minimum to the other, both the local force, given by *F*_*pp*_ in Eq. (1), and the substrate force, given by *F*_*s*_ in Eq. (1), depend on the position ${r}_{||}$ of the particle in the trap. A contraction of the defect line can occur when the local force is large enough to overcome the substrate force, *F*_*pp*_ > *F*_*s*_. For simplicity, consider ${F}_{pp\mathrm{,1}}^{c}$ and ${F}_{pp,3}^{c}$, which are the projections of the local forces acting on the particle parallel to the trap axis for contraction of the defect line at the 1-in or 3-in end, respectively. As shown schematically in Fig. 3, it is clear that at the beginning of the switching transition, ${F}_{pp\mathrm{,3}}^{c}\approx 2{F}_{pp\mathrm{,1}}^{c}$ since the repulsive force acting on the switching particle is produced by two particles at the 3-in end but by only one particle at the 1-in end. As a result, *v*_3_ > *v*_1_.

The local forces ${F}_{pp,\beta }^{c}$, where *β* = 1, 3, depend quadratically on the applied magnetic field, allowing us to write ${F}_{pp,\beta }={k}_{\beta }^{c}{B}^{2}$ with ${k}_{1}^{c} < {k}_{3}^{c}$. For small enough ${F}_{pp,\beta }^{c}$, there is a position ${\bar{r}}_{||}$ at which ${F}_{pp,\beta }^{c}({\bar{r}}_{||}) < {F}_{s}({\bar{r}}_{||})$. Writing ${F}_{{\rm{trap}}}={F}_{s}({\bar{r}}_{||})$, we see that when *B* is small enough, both *F*_*pp*,1_ and *F*_*pp*,3_ are smaller than *F*_trap_ and *v*_1_ = *v*_3_ = 0, giving a stable (S) defect line. When ${F}_{{\rm{trap}}}/{k}_{3}^{c} < {B}^{2} < {F}_{{\rm{trap}}}/{k}_{1}^{c}$, *v*_3_ > 0 but *v*_1_ = 0 as in Fig. 2(d), producing a one-sided slow contraction (SC3) state. For ${B}^{2} > {F}_{{\rm{trap}}}/{k}_{1}^{c}$, *v*_3_ > 0 and *v*_3_ > 0 as in Fig. 2(e), giving two-sided slow contraction (SC). There is an even higher critical value for *B* above which the local forces acting on the particles *within the defect line* exceed *F*_trap_, permitting the line to disintegrate via the nucleation of monopole-antimonopole couples.

### Effect of biasing force

**Figure 4:**
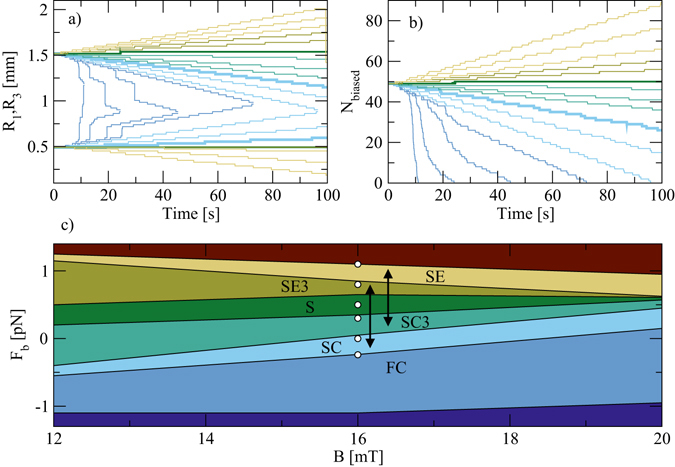
Biased systems. (**a**) The positions *R*_1_ (bottom lines) and *R*_3_ (top lines) of the ends of a defect line vs time in a sample with *B* = 16 mT for varied biasing forces *F*_*b*_ = −0.3, −0.27, −0.25, −0.24, −0.2, −0.1, 0 (thick light blue line), 0.1, 0.2, 0.3, 0.5 (thick green line), 0.7, 0.8, 0.9, 1.0, and 1.1, from left to right. (**b**) *N*_biased_, the number of vertices in the biased ground state, vs time in the same system for the same biasing forces as in panel (**a**), *F*_*b*_ = −0.3, … 1.1 from left to right. (**c**) Phase diagram as a function of *F*_*b*_ vs *B* showing the different phases of defect line contraction and expansion. Dark blue: Global nucleation of 1-in/3-in and biased ground state vertices (GN). Medium blue: Fast contraction with nucleation of 1-in/3-in vertex pairs along the defect line (FC). Light blue: Slow contraction on both ends of the defect line (SC). Light green: Slow contraction of only the 3-in end (SC3). Dark green: Stable defect string (S). Olive: Slow expansion of only the 3-in end (SE3). Yellow: Slow expansion on both ends of the line (SE). Red: Global nucleation of 1-in/3-in and biased ground state vertices (GN). The arrows indicate possible biasing force combinations that can be applied in order to generate a forward or backward ratcheting defect line. The series of white circles indicate the values of *F*_*b*_ = −0.25, 0, 0.3, 0.5, 0.8 and 1.1 at *B* = 16 *mT*.

If we apply a biasing force *F*_*b*_ along a diagonal direction, as shown in Fig. 1(a), we can change the energy balance between the ground state and biased ground state vertices. At sufficiently large ${F}_{b}={F}_{b}^{0}$, the biased and ground state vertices have the same energy so the defect line is stable and does not contract. For ${F}_{b} > {F}_{b}^{0}$, the biased state becomes energetically more favorable than the ground state and the defect line begins to grow. A very high biasing force causes defect lines to nucleate spontaneously and spread throughout the system until every vertex has switched to the biased state. In Fig. 4(a) we plot the time-dependent position of the 1-in and 3-in ends of a defect line at different biasing forces. For high *F*_*b*_, we find a fast contraction (FC) in which, in addition to the contraction of the line at each end, we observe spontaneous nucleation of 1-in/3-in vertex pairs along the line that speed up the contraction. At very large |*F*_*b*_|, we observe a global nucleation (GN) of 1-in/3-in pairs that spontaneously produce defect lines in the bulk which propagate through the system until the entire sample reaches a biased ground state. This process appears after 98 seconds in the *F*_*b*_ = 1.1 sample in Fig. 4(a), where *R*_3_ suddenly drops from *R*_3_ = 2 to *R*_3_ = 1.4. At larger values of *F*_*b*_, the nucleation process occurs more rapidly. In Fig. 4(b) we quantify the line contraction by plotting the total number *N*_biased_ of biased ground state vertices in the system. This measure shows the shrinking, stabilization, and growth of defect lines for different biasing forces, and can also capture the behavior of the system when spontaneous nucleation comes into play, either along the defect line in the case of fast contraction, or everywhere in the sample in the GN regime.

The interplay between the particle-particle interactions and the biasing force produces a rich phase diagram, shown in Fig. 4(c) as a function of *F*_*b*_ versus *B*. Consider the effects of ${F}_{pp\mathrm{,1}}^{c}$ and ${F}_{pp\mathrm{,3}}^{c}$ in the presence of a stabilizing biasing force *F*_*b*_. The 1-in end is stabilized when ${F}_{b} > {F}_{pp\mathrm{,1}}^{c}-{F}_{{\rm{trap}}}$. Thus, the SC3-SC transition follows the line ${F}_{b}={k}_{1}^{c}{B}^{2}-{F}_{{\rm{trap}}}$. Similarly, the 3-in end is stabilized when ${F}_{b} > {F}_{pp\mathrm{,3}}^{c}-{F}_{{\rm{trap}}}$, so the SC3-S transition can be described by ${F}_{b}={k}_{3}^{c}{B}^{2}-{F}_{{\rm{trap}}}$, keeping in mind that ${k}_{1}^{c} < {k}_{3}^{c}$.

If *F*_*b*_ is large enough, rather than merely stabilizing the defect line it can cause the line to grow. Figure 4(c) shows regimes of one-sided slow expansion (SE3) on only the 3-in end, as well as slow expansion (SE) on both ends of the defect line. We introduce ${F}_{pp\mathrm{,3}}^{e}={k}_{3}^{e}{B}^{2}$ and ${F}_{pp\mathrm{,1}}^{e}={k}_{1}^{e}{B}^{2}$, which are the forces acting on the particles that drive the *extension* rather than the contraction of the 3-in and 1-in ends, respectively. An elongation of the defect line on the 3-in side occurs when ${F}_{b} > {F}_{{\rm{trap}}}-{F}_{pp\mathrm{,3}}^{e}$, so that ${F}_{b}={F}_{{\rm{trap}}}-{k}_{3}^{e}{B}^{2}$ describes the S-SE3 transition. Similarly, ${F}_{b}={F}_{{\rm{trap}}}-{k}_{3}^{e}{B}^{2}$ describes the SE3-SE transition line. For extreme values of *F*_*b*_ in Fig. 4(c), the biasing force is so strong that the behavior cannot be described in terms of one-body motion. Instead, the whole sample switches to the biased state by global nucleation of 1-in/3-in vertex pairs and the spreading of defect lines (GN).

### Ratchet motion under an ac bias

**Figure 5:**
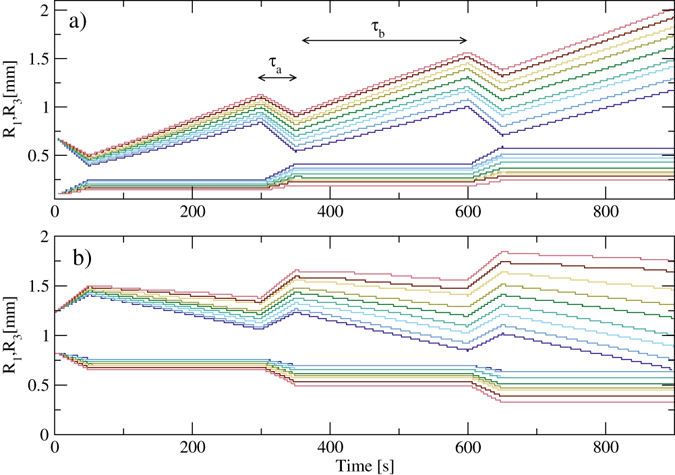
Ratcheting defect lines. *R*_1_ (bottom lines) and *R*_3_ (top lines), in mm, vs time in samples with *B* = 16 mT for alternating drive intervals with bias ${F}_{b}^{a}$ applied for *τ*_*a*_ = 50 s and ${F}_{b}^{b}$ applied for *τ*_*a*_ = 250 s during each cycle. (**a**) Forward ratchet effect for (${F}_{b}^{a}$, ${F}_{b}^{b}$) values of (−0.18, 0.76), (−0.16, 0.77), (−0.14, 0.78), (−0.12, 0.79), (−0.10, 0.8), (−0.08, 0.81), (−0.06, 0.82), (−0.04, 0.83) and (−0.02, 0.84), from blue to red. (**b**) Reverse ratchet effect for (${F}_{b}^{a}$, ${F}_{b}^{b}$) values of (0.96, 0.22), (0.98, 0.23), (1.0, 0.24), (1.02, 0.25), (1.04, 0.26), (1.06, 0.27), (1.08, 0.28), (1.1, 0.29) and (1.12, 0.3), from blue to red.

By tilting the sample back and forth over an appropriate range of angles, we can generate an ac external biasing force that causes the defect lines to oscillate by repeatedly growing and shrinking. If we allow the biasing force to switch instantaneously, or at least faster than typical defect speeds, between values ${F}_{b}^{a}$ and ${F}_{b}^{b}$, we can select pairs of biasing forces (${F}_{b}^{a}$, ${F}_{b}^{b}$) for which *v*_1_ ≠ *v*_3_, permitting the creation of a ratchet effect. Here we assume that the colloidal particles remain in contact with the trap potential at all times and are not pressed into or away from the trap potential by the angular acceleration produced by the mechanical tilting process. The magnitude of the angular acceleration at which this assumption breaks down depends on the viscosity of the surrounding fluid and the density of the colloidal particles. In Fig. 5 we show *R*_1_ and *R*_3_ versus time under an alternating biasing force where ${F}_{b}^{a}$ is applied for *τ*_*a*_ = 50 s and ${F}_{b}^{b}$ is applied for *τ*_*b*_ = 250 s per cycle. Here the defect line ratchets in the direction of the 3-in end through a wriggling motion that is composed of two simple phases. The biasing force ${F}_{b}^{a}$ places the sample in the SC regime where both ends of the line contract with *v*_3_ > *v*_1_. Then, under the biasing force ${F}_{b}^{b}$, the sample enters the SE3 regime where the line expands only on the 3-in end. As a result, over successive biasing force cycles the entire defect line translates in the direction of its 3-in end. It is also possible to choose the biasing forces in such a way that under ${F}_{b}^{a}$ the sample is in the SE regime, where both ends expand with *v*_3_ > *v*_1_, while under ${F}_{b}^{b}$ contraction occurs at only the 3-in end in the SC3 regime. Under these conditions, the defect line translates in the direction of its 1-in end, as shown in Fig. 5(b). By adjusting the timing of the expansion and shrinking drives (*τ*_*a*_ and *τ*_*b*_), we can slowly shrink, grow or maintain a constant defect line length as the line ratchets. This makes it possible to re-position defect segments inside the sample by varying an applied uniform external force.

## Discussion

We have shown that a defect line in a colloidal spin ice system contracts spontaneously at a rate which increases as the colloid-colloid interaction strength is increased. The line can be stabilized by the addition of a uniform global biasing force. It is possible to control the length and the position of the defect line by cycling this biasing force to create oscillations and defect movement through a ratchet effect. The ratcheting allows us to reposition defect line segments inside the sample to desired locations after nucleating them at the sample edge, making it possible to write information into the spin ice. If the uniform spin ice lattice were replaced by a specifically tailored landscape, it is possible to imagine the creation of logic gates and fan-out positions where defect lines can merge or split. Thus it could be possible to construct a device capable of storing and manipulating the information described by these defect lines through the creation of “defectronics” in spin ice that could be the focus of a future study building on defect line mobility and control in spin ices. Although we concentrate on magnetic colloidal particles, our results could also be applied to charge-stabilized colloidal systems with Yukawa interactions, for which it is possible to create large scale optical trapping arrays^23, 24^ and double-well traps^25^, and where biasing could be introduced by means of an applied electric field^26^. Compared to atomic spin ices, our colloidal spin ice has relatively low density and, if it were used for information storage, would have relatively low write speeds. If the processes we model here can be introduced into a magnetic spin ice material, it would be possible to create a very high density information storage unit surpassing currently available densities by several orders of magnitude.

## Methods

### Numerical simulation details

Using Brownian dynamics, we simulate an experimentally feasible system^15^ of paramagnetic colloids placed on an etched substrate of pinning sites. The spherical, monodisperse particles have a radius of *R* = 5.15 *μ*m, a volume of *V* = 572.15 *μ*m^3^ and a density of *ρ* = 1.9 × 10^3^ kg/m^3^. They are suspended in water, giving them a relative weight of *W* = 5.0515 pN. Gravity serves as a pinning force for the particles placed in the etched double-well pinning sites and also generates a uniform biasing force *F*_*b*_ = *W* sin(*α*) on all particles when the entire sample is tilted by *α* degrees. Typically, *α* ~ 10°. The double well pinning sites [Fig. 1(a)] representing the spins in the spin ice are etched into the substrate in the 2D square spin ice configuration [Fig. 1(c,d)] with an interwell spacing of *a* = 29 *μ*m. Each pinning site contains two minima that are *d* = 10 *μ*m apart. We place one particle in each pinning site, which can be achieved experimentally by using an optical tweezer to position individual particles. The pinning force *F*_*s*_ acting on the particle is represented by a spring that is linearly dependent on the distance from the minimum, so that ${F}_{s\perp }=2kW{\rm{\Delta }}{r}_{\perp }$, where *k* = 1.2 × 10^−4^ nm^−1^ is the spring constant, and ${\rm{\Delta }}{r}_{\perp }$ is the perpendicular distance from the particle to the line connecting the two minima. When the particle is inside one of the minima, ${F}_{s||}=2kW{\rm{\Delta }}{r}_{||}$, where ${\rm{\Delta }}{r}_{||}$ is the distance from the particle to the closest minimum along the line connecting them, while when the particle is between the minima, ${F}_{s||}=8h/{d}^{2}W{\rm{\Delta }}{r}_{||}$, where *h* = 0.87 *μ*m is the magnitude of the barrier separating the minima and ${\rm{\Delta }}{r}_{||}$ is the distance between the particle and the barrier maximum parallel to the line connecting the two minima. During the simulation, the particles are always attached with these spring forces to their original pinning sites.

The inter-particle repulsive interaction arises from the magnetization induced by the external magnetic field that is applied perpendicular to the pinning site plane. Each particle acquires a magnetization of *m* = *BχV*/*μ*_0_, where *B* is the magnetic field in the range of 0 to 30 mT, *χ* = 0.061 is the magnetic susceptibility of the particles, and *μ*_0_ = 4*π* × 10^5^ pN/A^2^ is the magnetic permeability of vacuum. The repulsive force between particles is given by *F*_*pp*_ = 3 *μ*_0_ *m* ^2^/(2*πr* ^4^), and since it has a 1/*r* ^4^ dependence in a 2D system we can safely cut it off at finite range. We choose a very conservative cutoff distance of *r*_*c*_ = 60 *μ*m to include next-nearest neighbor interactions (even though they are negligibly small).

During the simulation we solve the discretized Brownian dynamics equation:

1 $$\frac{1}{\mu }\frac{{\rm{\Delta }}{x}_{i}}{{\rm{\Delta }}t}=\sqrt{\frac{2}{D{\rm{\Delta }}t}}{k}_{B}TN\mathrm{[0},\mathrm{1]}+{F}_{pp}^{i}+{F}_{s}^{i}+{F}_{b}^{i}$$

where *F*_*pp*_, *F*_*s*_ and *F*_*b*_ are the previously described particle-particle, particle-substrate, and biasing forces, *k*_*B*_ *T* = 4.047371 pN · nm is the thermal energy, *D* = 7000 nm^2^/s is the diffusion constant, *μ* = *D*/(*k*_*B*_ *T*) is the mobility of the particles, *N*[0, 1] is a Gaussian distributed random number with mean of 0 and standard deviation of 1, and Δ = 1 ms is the size of a simulation time step.
